# Differential Salt-Induced Dissociation of the p53 Protein Complexes with Circular and Linear Plasmid DNA Substrates Suggest Involvement of a Sliding Mechanism

**DOI:** 10.3390/ijms16023163

**Published:** 2015-01-30

**Authors:** Peter Šebest, Marie Brázdová, Miroslav Fojta, Hana Pivoňková

**Affiliations:** 1 Institute of Biophysics, Academy of Sciences of the Czech Republic, v.v.i., Královopolská 135, Brno CZ-612 65, Czech Republic; E-Mails: besta@ibp.cz (P.S.); maruska@ipb.cz (M.B.); 2 Central European Institute of Technology, Masaryk University, Kamenice 753/5, Brno CZ-625 00, Czech Republic

**Keywords:** tumor suppressor p53, protein-DNA binding, association, dissociation, stability, salt concentration

## Abstract

A study of the effects of salt conditions on the association and dissociation of wild type p53 with different ~3 kbp long plasmid DNA substrates (supercoiled, relaxed circular and linear, containing or lacking a specific p53 binding site, p53CON) using immunoprecipitation at magnetic beads is presented. Salt concentrations above 200 mM strongly affected association of the p53 protein to any plasmid DNA substrate. Strikingly different behavior was observed when dissociation of pre-formed p53-DNA complexes in increased salt concentrations was studied. While contribution from the p53CON to the stability of the p53-DNA complexes was detected between 100 and 170 mM KCl, p53 complexes with circular DNAs (but not linear) exhibited considerable resistance towards salt treatment for KCl concentrations as high as 2 M provided that the p53 basic *C*-terminal DNA binding site (CTDBS) was available for DNA binding. On the contrary, when the CTDBS was blocked by antibody used for immunoprecipitation, all p53-DNA complexes were completely dissociated from the p53 protein in KCl concentrations ≥200 mM under the same conditions. These observations suggest: (a) different ways for association and dissociation of the p53-DNA complexes in the presence of the CTDBS; and (b) a critical role for a sliding mechanism, mediated by the *C*-terminal domain, in the dissociation process.

## 1. Introduction

Tumor suppressor protein p53 is a multifunctional protein that plays a critical role in the cell defense against malignant transformation, maintaining of genomic integrity, control of cell proliferation, apoptosis [1] and senescence, and its mutations (mainly within the central DNA-binding domain) can be found in more than 50% of all human tumors. Protein p53 controls expression of target genes by either activation or inhibition of p53-responsive promoters [2]. It acts as a stress-induced transcription factor and its functions are closely connected with various modes of DNA binding. The p53 protein binds DNA sequence specifically via its core domain to target sequences consisting of two copies of a motif 5'-RRRC(A/T)(T/A)GYYY-3' (p53CON) separated by 0–13 bp [3,4]. This binding is essential for p53 to function as a transcription factor and it is usually lost in cancer-related p53 mutants [5,6]. Further, p53 binds DNA sequence non-specifically via its basic *C*-terminal domain, which is essential for structure-selective DNA binding, such as preferential interactions with negatively or positively supercoiled DNA [5,6,7,8,9,10], non-B structures stabilized by negative DNA supercoiling, such as cruciform [11,12,13], and to chemically modified/damaged DNA [14,15]. The p53 *C*-terminal domain is known to cooperate with the core domain to recognize efficiently the p53CON response elements [4]. It is essential for the protein sliding along DNA duplex while searching for the p53CON [16,17,18,19,20,21].

In this paper we present a study of dissociation of the p53 protein complexes with different circular and linear plasmid DNA substrates by exposure to increased salt concentrations. Using a magnetic beads-based immunoprecipitation assay, we show that the stability of the p53-DNA complexes towards salt induced dissociation is strongly influenced by presence or absence of free ends in the bound DNA, and by availability of the basic *C*-terminal DNA binding site.

## 2. Results and Discussion

### 2.1. Effect of Salt Concentration on p53-DNA Association

It has been well established that DNA-protein interactions, including DNA binding by the p53 protein, are affected by salt concentration. In general, at lower salts relative strong non-specific binding to duplex DNA, involving a significant electrostatic contribution, is observed while at moderate salt conditions (more or less within the physiological region of monovalent ions concentration) the non-specific interactions are suppressed while sequence- or some modes of structure-specific binding are retained [4,20,22,23]. Here we focused on the influence of salt condition on formation of wild type p53 complexes with DNA and salt induced dissociation of p53 complexes with ~3 kbp long plasmid DNA substrates in various DNA conformations (supercoiled, relaxed circular and linear), containing or lacking a specific p53 binding site, p53CON (so far the majority of the association and stability studies were performed with short oligonucleotides in their linear forms [24]).

**Figure 1:**
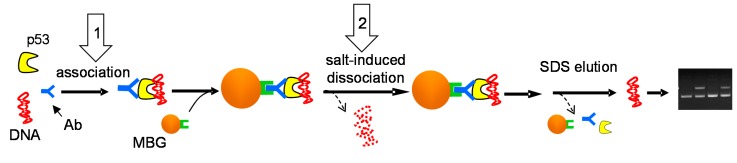
Scheme of the magnetic beads-based immunoprecipitation (MBIP) assay of the p53–DNA binding and dissociation. An antibody (Ab) is mixed with the p53 protein in solution, followed by addition of a DNA substrate. Then the Ab-p53-DNA complex is captured at the protein G-coated magnetic beads (MBG) via the Ab-protein G linkage. After repeated washing and/or incubation under different salt conditions, DNA remaining at the beads is released due to exposure to SDS and elevated temperature and samples loaded on agarose gel. In steps marked with arrows 1 and 2, effects of salt concentration on formation or dissociation of the p53-DNA complexes, respectively, were inspected (for more details see text).

**Figure 2:**
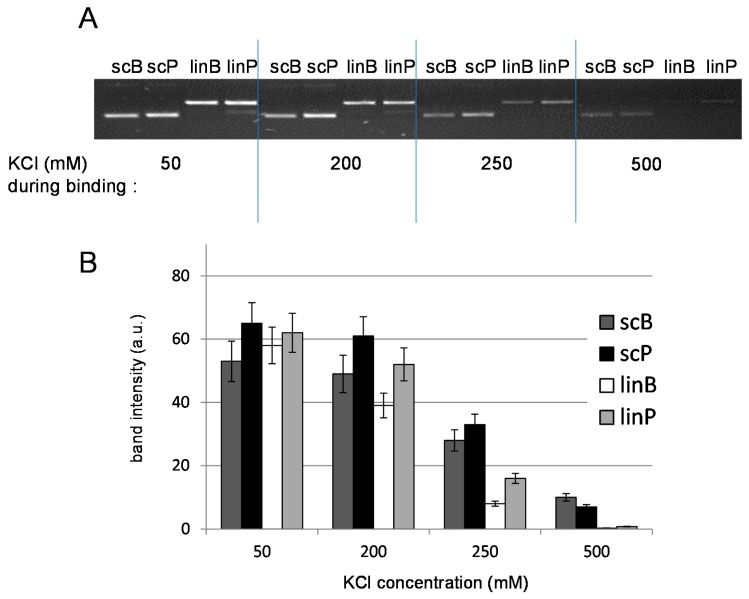
Effects of KCl concentration on the formation of the p53-DNA complexes. (**A**) Agarose gel electrophoresis of recovered DNA after the MBIP assay (Figure 1); and (**B**) Histograms showing relative intensities of bands of the recovered sc and lin forms of pBSK and pPGM1 plasmids (see legend in the figure). (DO-1)-p53-DNA complexes were formed in solution containing the given KCl concentration and captured at the MBG from the same medium. Beads were then washed by binding buffer containing 50 mM KCl and the bound DNA was eluted by SDS treatment. For more details, see Results and Discussion, and Experimental Section.

Effects of increased salt concentration on the stability of the p53–DNA complexes were investigated using the MBIP technique (Figure 1), which has recently proved useful in studies of relative binding affinity of the p53 protein to structurally distinct DNA substrates [10,13,25]. Here we first inspected effects of KCl concentration present in the samples during formation of the p53-DNA complex. The p53 protein was mixed with DO-1 antibody (Ab) and a DNA substrate in solution containing KCl at the given concentration, followed by incubation to allow formation of the Ab-p53-DNA complexes, which were then captured at magnetic beads coated with protein G (MBG) (Figure 1). After washing in 50 mM KCl, DNA was eluted from the beads by heating in medium containing 1% SDS, and the amount of DNA recovered from the beads in the latter step was analyzed by agarose gel electrophoresis (Figure 2A) and densitometry (Figure 2B). In the absence of either p53 or the antibody, no DNA was recovered (not shown here but reported previously [10,26]), confirming that non-specific adsorption of DNA or the p53-DNA complexes at the MBG does not contribute significantly. When the Ab-protein-DNA complexes were formed and bound to the beads in 50 mM KCl, the recovered amounts of all tested DNAs (supercoiled pBSK (scB, without p53CON) and pPGM1 (scP, with p53CON) as well as linear forms of the same plasmids (linB and linP, respectively)) were comparable (varying within the experimental error, see histograms in Figure 2B). In 200 mM KCl the amount of bound linB was decreased by 25%–30%, while the amount of linP decreased less significantly and amounts of recovered scDNAs were practically unchanged. When the KCl concentration was increased to 250 mM, p53 binding to scDNAs was decreased to about 50%, binding of the protein to linP to 25% and binding to linB to 10%–15%. In 500 mM KCl, the p53-scDNA binding was suppressed to 10%–20%, as compared to binding in 50 mM KCl, and recovered amounts of linDNAs were at the limit of detection in agarose gels. In general, the above results revealed significant inhibitory effects of KCl at concentrations between 200 and 500 mM on the *association* of the p53 protein with diverse DNA substrates.

**Figure 3:**
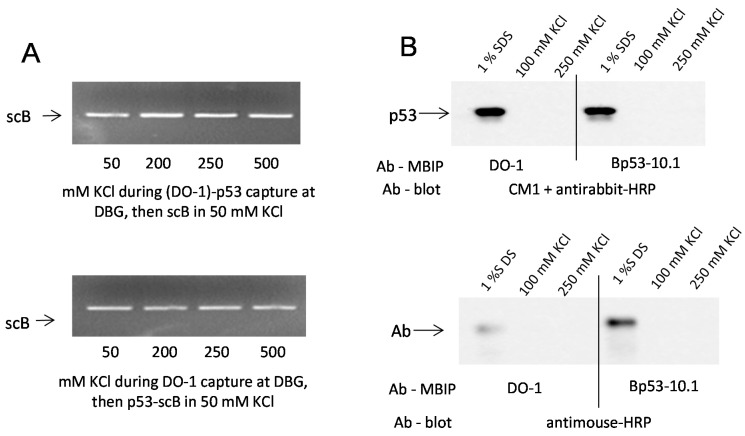
Effects of KCl concentration on (protein G)-Ab or Ab-p53 bonds. (**A**) **Top**: (DO-1)-p53 immune complex was formed in solution and captured at the MBG from binding buffer containing the given KCl concentrations, followed by binding of scB DNA from 50 mM KCl; **Bottom**: DO-1 Ab was immobilized at the MBG from the given KCl concentration; p53-scB DNA complex was formed in solution containing 50 mM KCl and from the same medium it was captured at the (DO-1)-modified MBG. In both cases, the beads were then washed in 50 mM KCl, DNA was released by SDS treatment and analyzed by agarose gel electrophoresis; and (**B**) **Top**: Western blot analysis of the p53 protein after treatment of MBG with immunoprecipitated (DO-1)-p53 or (B-10)-p53 complexes with 1% SDS, 100 or 250 mM KCl. The samples were resolved by 12.5% SDS-polyacrylamide electrophoresis. Proteins from the gels were transfered onto nitrocellulose membranes. The p53 protein was detected using rabbit polyclonal CM1 antiserum and antirabbit-HRP conjugate; **Bottom**: as above but antimouse-HRP conjugate was used to detect the anti-p53 antibodies.

Control experiments were performed to prove that exposure to elevated salt actually influences p53-DNA binding, while bonds between protein G and the beads, between the Ab and protein G or between p53 and the Ab are not significantly influenced (Figure 3). First, (DO-1)-p53 immune complex was formed in the absence of DNA in solution containing different KCl concentrations and from the same media it was captured at the MBG, followed by incubation of the beads in scB DNA solution containing 50 mM KCl (Figure 3A, top). Second, the DO-1 Ab was immobilized at the MBG from the given KCl concentration and wtp53-scB DNA complex was formed in solution containing 50 mM KCl, from which it was subsequently captured at the (DO-1)-modified MBG (Figure 3A, bottom). In both cases, the beads were then washed in 50 mM KCl, the DNA was eluted by SDS treatment and analyzed by agarose gel electrophoresis. Results clearly demonstrate that the amount of captured/recovered DNA did not depend on KCl concentration from which either the Ab-p53 complex (Figure 3A, top) or the Ab (Figure 3A, bottom) was immobilized at the beads.

### 2.2. Effects of DNA Structure, Presence of p53CON and Availability of CTDBS on Salt-Induced Dissociation of the p53-DNA Complexes

**Figure 4:**
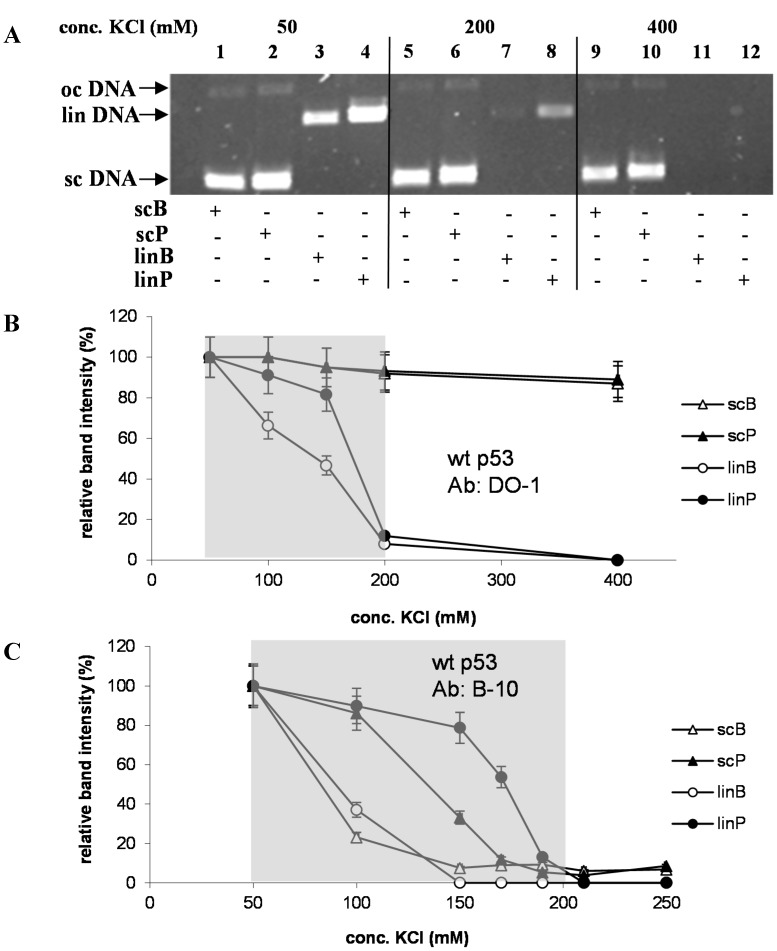
(**A**) Agarose gel electrophoresis of DNA recovered from MBG after incubation of the DO-1-p53-DNA complex at the beads to 50, 200 or 400 mM KCl (see legend on the top) for 30 min at 10 °C (step marked in Figure 1 by arrow 2) followed by the SDS treatment. DNA substrates used are specified on the bottom: scB and scP, supercoiled pBSK and pPGM1 plasmids, respectively, both with native superhelix density (σ); linB and linP, the same but linearized plasmids (see Experimental Section); (**B**) Dependence of relative fractions of DNAs resisting salt treatment on KCl concentration to which immunoprecipitated (DO-1)-p53-DNA complex was exposed (conditions as in A). Data obtained from densitometric scanning of ethidium-stained agarose gels; value obtained for 50 mM KCl was taken as 100% for each DNA substrate; and (**C**) As in B but antibody B-10 was used instead of DO-1. Shaded areas in (**B**) and (**C**) indicate region of KCl concentrations between 50 and 200 mM.

**Figure 5:**
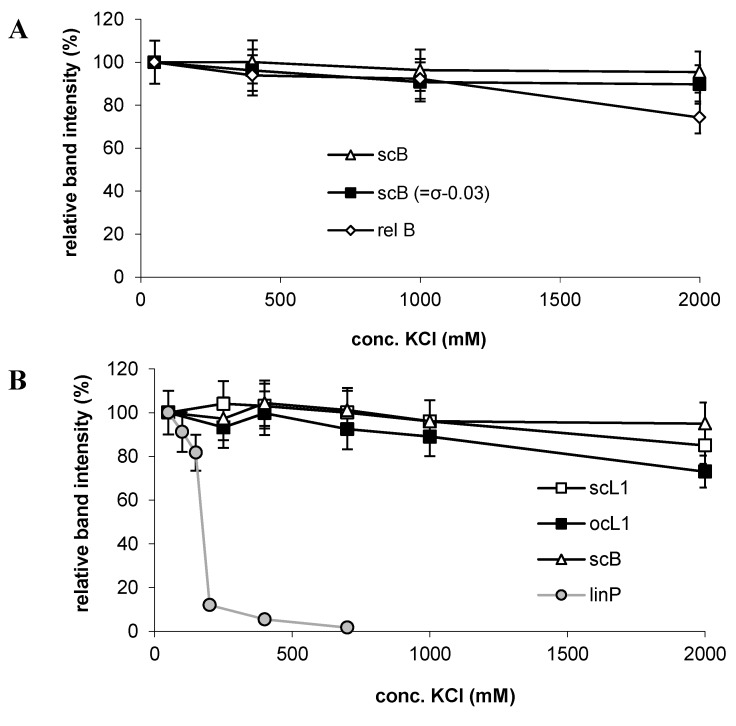
Dependence of relative fractions of DNAs resisting salt treatment on KCl concentration to which the immunoprecipitated (DO-1)-p53-DNA complexes were exposed. Analogous experiments as in Figure 2B with different DNA substrates: (**A**) pBSK plasmid: sc with native σ (*i.e.*, scB), sc with reduced σ (−0.03) and in covalently closed relaxed form (rel); and (**B**) sc pL1, native σ (scL1), open circular pL1 (ocL1), sc pBSK, native σ (scB); data for linP taken for comparison from Figure 2B. Other conditions as in Figure 4.

Recently we found and analytically utilized [26] a remarkable stability of the p53 protein binding to supercoiled (sc) DNA in relatively high salt: practically no dissociation of p53-scDNA complex was observed upon exposure to 400 mM KCl for 5 min, while complexes of p53 with linear (lin) DNAs were completely dissociated under the same conditions. To study salt-induced dissociation of the p53-DNA complexes in more detail, Ab-p53-DNA complexes were prepared in solution containing 50 mM KCl and then immobilized at MBG (Figure 1), subsequently followed by incubation of the beads in solution containing different concentrations of KCl, a short washing in 50 mM KCl and elution of DNA remaining bound by the p53 protein after the salt treatment by heating in 1% SDS as above. Figure 4A shows a comparison of amounts of scB, scP, linB and linP DNAs, co-immunoprecipitated with the DO-1 Ab and recovered after treatment in 50, 200 and 400 mM KCl. Both scDNAs did not exhibit significant decrements of the band intensity due to increase of KCl concentration from 50 to 200 mM and only slight decrease for 400 mM KCl; strikingly, p53 complexes with scDNAs exhibited a considerable stability, even at 2 M KCl (shown for scB in Figure 5). On the contrary, amounts of recovered linDNAs decreased in 200 mM KCl to about 10% of values observed in 50 mM KCl for each of the linDNA substrates (albeit absolute amounts of the recovered sequence-specifically bound linP were remarkably higher than those of the non-specific linB, Figure 4A), and practically disappeared after treatment in 400 mM KCl. Significant differences between stability of the p53-linB and p53-linP complexes were observed upon exposure to 100 and 150 mM where significant positive effect of the p53CON in linP took place (Figure 4B). These findings are in a good agreement with previous general conclusions based on association studies with short DNA (to 50 bp) by fluorescence anisotropy [24].

In the next experiments the antibody B-10 was used for the MBIP instead of DO-1. While DO-1 recognizes an epitope in the p53 *N*-terminal domain (mapping to aa 21–25), leaving its *C*-terminal DNA binding site (CTDBS) available for interaction with DNA, the B-10 blocks the CTDBS (mapping to aa 375–379), thus suppressing its high-affinity supercoil-selective DNA binding and activating it for the sequence-specific DNA binding [7,8]. In apparent agreement with these features, both sequence non-specific DNAs regardless of their superhelicity, linB and scB, were almost completely dissociated from the (B-10)-p53 immune complex in KCl at concentrations of 150 mM and higher (Figure 4C). A comparison of the behavior of scP and linP suggests an apparently higher stability of the sequence-specific p53 complex with linDNA than with scDNA in 150 mM KCl and higher. This may be rather surprising with respect to the earlier established stimulating effect of DNA negative superhelicity on p53 binding to certain p53CONs [13], including that present in the pPGM1 plasmid [12]. Nevertheless, considering earlier observed certain preference of the p53 core DNA binding domain for supercoiled DNA without the involvement of the CTDBS [7,27], one can expect that, in scP, the DNA superhelicity may partially disfavor the (B-10)-p53 immune complex to find the p53CON. Thus, two mutually competing binding modes may be involved in the (B-10)-p53-scP complex: one in which the protein is bound to p53CON (partly resisting the salt treatment between 100 and 150 mM KCl (Figure 4C) and the other in which p53 interact with scDNA anywhere outside the specific sequence (which is more prone to the salt-induced dissociation when the CTDBS is unavailable, compare with scB, Figure 4B).

A control experiment was performed (Figure 3B) to test the stability of protein G-Ab and Ab-p53 linkage in the region of KCl concentrations where dissociation of p53 complexes with lin DNAs was observed. MBG with immunoprecipitated (DO-1)-p53 or (B-10)-p53 complexes were treated with 1% SDS, 100 mM KCl or 250 mM KCl, followed by SDS-polyacrylamide electrophoresis and Western blot analysis of the eluate. The p53 protein was detected using rabbit polyclonal CM1 antiserum and antirabbit-HRP conjugate (Figure 3B, top); antimouse-HRP conjugate was used to detect the DO-1 and B-10 Abs (Figure 3B, bottom). These experiments confirmed that during the salt treatment, detectable amounts of neither p53 nor Ab were released from the beads. Both species were detected only after heating in 1% SDS; hence, the observed loss of recovered linear DNAs due to treatment in 100–200 mM KCl is due to dissociation of DNA from p53.

### 2.3. Circular Shape of p53-Bound DNA Molecule rather than DNA Superhelicity Renders the (DO-1)-p53-DNA Complexes Stability in High Salts

Previous literature [5,7,8,9,10,11,12,27,28,29] revealed the DNA superhelicity to have a remarkable stimulating effect on p53-DNA binding, both through a sequence-independent supercoil-selective mode [7,8,10,27] and through negative superhelicity-induced structural rearrangement of p53CONs [11,12,13,28,29] or other putative p53-specific binding sites [5,30]. In general, these binding modes require involvement of the p53 CTDBS. Hence it was apparently rational to assume that it was the DNA superhelicity that was rendering the considerable resistance of the (DO-1)-p53-scB and (DO-1)-p53-scP complexes towards treatment in relatively high salt concentrations. Moreover, our previous study [10] has shown that the affinity of the p53 protein to scDNA increases with the number of negative or positive DNA superturns, and that the protein affinity to the relaxed covalently closed circular DNA lacking the p53CON is comparable to the affinity of the same protein to linDNA. Therefore, next experiment in this study was focused on how negative superhelicity level of the pBluescript DNA influences the stability of corresponding (DO-1)-p53-DNA complexes towards the salt-induced dissociation (Figure 5A). When the scB superhelix density (σ) was decreased from native (around −0.06) to σ = −0.03, no change in the stability of the (DO-1)-p53-scB complex was observed in the region of KCl concentration between 50 mM and 2 M (Figure 5A). Even when scB was completely topoisomerase-relaxed (σ~0, relB), no significant increase in the relative dissociation level was detected in KCl concentrations up to 1 M, and after treatment in 2 M KCl, the amount of recovered DNA decreased by about 20%, compared to scB with native σ. There was thus a striking contrast between the behavior of relB (remaining bound to p53 upon treatment in mol∙L^–1^ levels of KCl) and linB (being almost completely dissociated from the protein in ~200 mM KCl), in contrast to the expected similarity.

Since the relatively strong increase of the KCl concentration from 50 mM (in which the p53-relDNA complex was prepared) to 1–2 M can induce changes in the global DNA conformation, than could be compensated by formation of certain number of superhelix turns [31,32] that hypothetically might consequently have stabilized the p53-DNA complex, we performed analogous experiment with a nicked (open) circular DNA substrate. Such DNA molecule is, owing to free rotation within the single strand break, inherently relaxed regardless of conformation alterations of the DNA double helix induced by a change of external conditions. For this purpose we used plasmid pL1 [33,34] containing a site in which a single strand break per DNA molecule can be introduced by a nicking enzyme *Nb.BbvCI* [35]. Thus prepared open circular DNA of the pL1 plasmid (ocL1) was bound by the (DO-1)-p53 immune complex and subjected to the salt treatment at the MBG surface in the same way as above. Results in Figure 5B indicate a considerable stability of the p53-scL1 complex, showing only slightly increased dissociation level in 2 M KCl, when compared to scB and scL1. The behavior of ocL1 thus resembles that of the (sc or topoisomerase-relaxed) covalently closed circular DNA and strikingly contrasts with that of linDNA.

Taken together, these comparative studies suggest that the DNA superhelicity itself (in various previous studies established as a feature offering high-affinity p53 binding structural motifs [5,7,8,9,10]), need not necessarily be the principal player standing behind the remarkable stability of the (DO-1)-scDNA-p53 in high salt conditions, but rather it is the circular shape of DNA substrates (*i.e*., absence of DNA molecule ends) rendering the resistance of the corresponding nucleoprotein complexes towards the salt treatment. Other possibilities can be excluded considering common and distinct features among different DNA substrates used in these experiments: (a) presence of p53CON in linDNA stabilizes the corresponding p53-DNA complex only in KCl concentrations lower than about 200 mM; (b) an essential role for the single strand break as the primary p53-DNA complex-stabilizing entity in ocL1 is improbable because no such site is present in the covalently closed circular DNA substrates (including topoisomerase-relaxed DNA) exhibiting practically the same behavior (especially when compared to linDNA).

### 2.4. Possible Mechanism of Salt-Induced Dissociation of the p53-DNA Complexes

**Figure 6:**
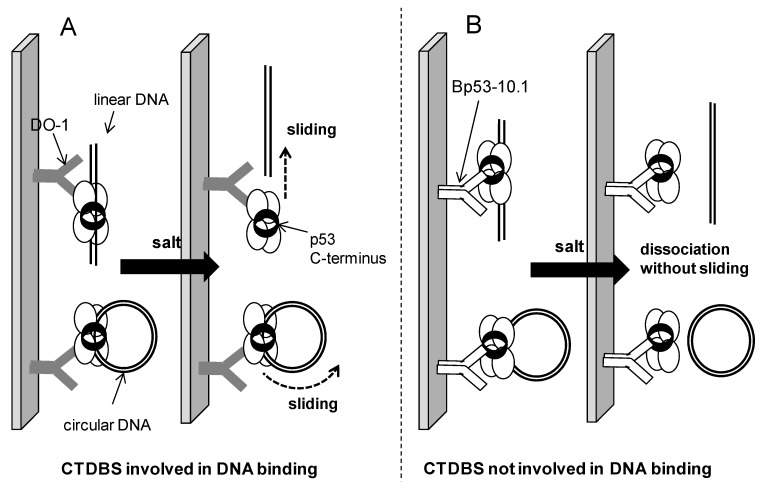
Scheme of possible mechanisms of dissociation of the p53 complexes with linear or circular DNAs for situations when the p53 CTDBS (*C*-terminal DNA binding site) either is or is not involved. (**A**) In immune complexes with the DO-1 Ab, the CTDBS is available for interaction with DNA. The considerable stability of the corresponding p53 complexes with circular DNA substrates can be attributed to sliding (linear diffusion) mechanism as the major contribution of the dissociation process (even in high salt, the protein remains bound to DNA, sliding freely along it; linDNA can easily slide-out from the complex while the “endless” circular DNA cannot); and (**B**) When the CTDBS is blocked by the B-10 Ab, the (sequence non-specific) sliding mechanism does not take place, resulting in no differences between sc and lin DNA upon salt treatment.

The higher stability of the sequence-specific p53-DNA complexes between ~100 and ~170 mM salt, as compared to non-specific DNA binding by the same protein, was reported previously by various authors [4,22,23,36] using short (several tens of bp) oligonucleotide targets. In this study we observed a reduced dissociation of linP and partly scP in the relevant KCl concentration region from the p53 immune complexes in the same salt concentration region, compared to analogous complexes with DNA lacking the p53CON, suggesting a contribution of the sequence-specific interaction to the p53-DNA stability within the ~3 kbp-long plasmid DNA substrates. Apart from these p53CON-dependent effects, two factors have been identified that critically influence the dissociation processes in elevated salt concentration regardless of the presence or absence of the p53 cognate site: (a) circularity *vs.* linearity (*i.e*. closed *vs.* open shape) of the DNA molecules; and (b) availability of the p53 CTDBS for interaction with DNA (which is preserved in the DO-1 but not B-10 immune complex). It has been established that the full length p53 protein possessing intact *C*-terminal domain uses linear diffusion (sliding) mechanism along DNA to search for specific binding sites [16,17,18,19,20], while the p53 core domain without involvement of the protein *C*-terminus rather uses “hopping” mechanism (involving repeated association/dissociation events) for the same purpose [19,21]. It can be expected that the same respective phenomena take part during the p53-DNA complexes dissociation. As depicted in Figure 6A, if the sliding mechanism is involved in dissociation of DNA molecule from the surface-attached (DO-1)-p53 immune complex, there is a simple way for a linear DNA to leave the complex by sliding-out. On the other hand, any circular DNA will never dissociate from the protein solely by this sliding. Even when certain structural motifs in the circular DNAs (such as cross-overs of double helices [7,10] or conformational constraints resulting from considerable double-helix curvature at the superhelix apices in scDNA (analogous effects of DNA conformation on protein sliding have recently been studied theoretically using topologically constrained small DNA circles [37]) or single strand break in ocDNA) may represent obstacles for a smooth movement of the protein-bound DNA molecule, the principal difference between a linear and a circular DNA, *i.e*., presence or absence of the double helix end at which the DNA can dissociate form the complex, is preserved. On the contrary, in the (B-10)-p53 immune complex the CTDBS of the protein cannot contribute to the protein-DNA interaction and mediate the sliding process, and in accordance with the literature data, the p53 protein bound to DNA only via its core domain can dissociate from the complex at any site (Figure 6B). Accordingly, no significant differences between linear and circular DNA substrates under dissociation-inducing conditions are observed.

## 3. Experimental Section

### 3.1. DNA Samples

Supercoiled (sc) plasmids pBSK(−), pL1 (both not containing p53CON) and pPGM1 (containing a p53CON AGACATGCCTAGACATGCCT) were isolated and purified as described earlier [38]. Linear (lin) DNAs were prepared by *SmaI* (New England BioLabs, Beverly, MA, USA) cleavage of the pBSK(−) and pPGM1 plasmids. Open circular (oc) DNA of the pL1 plasmid (derived from the single-*BbvCI* site plasmid described in Gowers *et al*. [33] and kindly donated by R.P. Bowater (University of East Anglia, Norwich, UK) was prepared by treatment with the DNA nicking mutant of *BbvCI* restrictase, *Nb.BbvCI* (New England BioLabs) [35]. Relaxed (rel) covalently closed circular DNA and scDNA with reduced superhelix density were prepared using wheat germ *topoisomerase I* (Promega, Madison, WI, USA) and/or ethidium bromide (Sigma, St. Louis, MO, USA) according to [39]*.*

### 3.2. Monoclonal Antibodies

Murine anti-p53 monoclonal antibodies (mAbs) DO-1 (mapping to aa 21–25 in the p53 *N*-terminus) and Bp53-10.1 (in this paper, abbreviation B-10 is used) (mapping to aa 375–379 in *C*-terminus) [40] were provided by Bořivoj Vojtěšek (Masaryk Memorial Cancer Institute, Brno, Czech Republic).

### 3.3. Preparation of Protein p53

Human full-length wild-type p53 protein was expressed in *Escherichia coli* BL21/DE3 cells and purified as described previously [7,8]. Protein concentration was determined densitometrically from Coomasie Blue R-250 stained gels using bovine serum albumine as a standard.

### 3.4. Preparation of the p53 Immune Complexes and p53-DNA Binding

The p53 immune complexes were prepared by mixing of DO-1 or B-10 antibody with the protein at a molar ratio of 8:1 in binding buffer (50 mM KCl if not stated otherwise, 5 mM Tris, 2 mM DTT and 0.01% Triton X-100, pH 7.6), followed by a 30-min incubation on ice. Then, 200 ng of the given DNA were mixed with the given immune complex (p53 tetramer/DNA molar ratio was 2.5:1) and incubated in the binding buffer for 30 min on ice.

### 3.5. Immunoprecipitation of the p53-DNA Complexes with Magnetic Beads (MBIP Assay)

Magnetic beads (12.5 μL of the stock suspension per sample) coated with protein G (MBG; Dynal^®^, Invitrogen, Carlsbad, CA, USA) were washed three times with 150 μL of the binding buffer. The beads were separated from the supernatant using magnetic particle concentrator. The binding reaction mixture was added and incubated with the beads for 30 min at 10 °C whilst shaking mildly. Then, the beads with bound immune complexes were incubated three times for 3 min at 10 °C in binding buffers containing different concentrations of the KCl. After washing, DNA was released from the beads by heating at 65 °C in 1% SDS for 5 min and electrophoresed in agarose gel.

### 3.6. Agarose Gel Electrophoresis

DNA recovered from the beads was separated in 1.3% agarose gel containing 1× Tris/acetate/EDTA buffer, pH 7.9. Gels were stained with ethidium bromide and photographed.

## 4. Conclusions

Results presented in this paper extend the insight into mechanism of processes of association and dissociation of the tumor suppressor p53 protein with long DNA molecules, particularly the effects of increased ionic strength on dissociation of the p53-DNA complexes immunoprecipitated at magnetic beads. To our best knowledge this is the first experimental study of the salt-dependent stability of the p53 complexes with several kbp-long plasmid DNA substrates in their circular and linear forms (a recent theoretical study [37] of salt-dependent protein sliding along DNA has used small, ≤100 bp DNA circles to model conformational constraints of the DNA double helix and their effects on the sliding process, rather than to study effects of free DNA ends on the association/dissociation process). In our experiments, salt concentrations above 200 mM KCl present in the medium during formation of the p53-DNA complex strongly affected binding (association) of the (DO-1)-p53 to any plasmid DNA substrate (Figure 2). In 250 mM KCl, contributions from both sequence-specific binding to a p53CON in linDNA and supercoil-selective binding was observed, while in 500 mM KCl only scDNAs exhibited significant but strongly reduced binding (compared to KCl concentration ≤100 mM). Dramatically different and surprising results were obtained when dissociation of (DO-1)-p53-DNA complexes formed in 50 mM KCl, induced by increased salt concentrations, was studied. While contribution from the p53CON to the stability of DNA complexes with p53 was detected between 100 and 170 mM KCl in agreement with expectations, (DO-1)-p53 complexes with circular DNAs exhibited considerable resistance towards salt treatment for KCl concentrations as high as 2 M. On the contrary, when B-10 antibody that (unlike DO1) blocks the p53 *C*-terminal DNA binding site was used in the MBIP procedure, any DNA was, under the same conditions, completely dissociated from the p53 protein in KCl concentrations ≥200 mM. These observations suggest that (a) the p53-DNA association process, at least when the p53 CTDBS is involved, is affected by high salt concentrations in a different way than dissociation. While higher KCl concentrations apparently prevent the protein to come into a close contact with DNA (possibly due to attenuation of electrostatic contribution from the basic CTDBS), once the protein-DNA surface adopts a proper configuration (at lower salt), the resulting complexes with circular DNAs exhibit the considerable resistance towards dissociation; (b) At the same time the striking difference between apparent stabilities of the (DO-1)-p53 complexes with circular *vs.* linear DNAs can be explained by a sliding mechanism of dissociation (Figure 6A) in the case of involvement of the CTDBS in the protein-DNA interaction. The ability of the p53 protein to remain associated with long non-specific DNA loops (domains) under physiological conditions is supposed to contribute to the efficiency of searching for its cognate sites. On the other hand, the striking stability of the (DO-1)-p53 complexes with circular DNA in salt concentrations far above physiologically relevant values can be exploited analytically for fishing-out, separation or fractionation proteins binding DNA in different modes.
